# Reviews and Book Notices

**Published:** 1879-04

**Authors:** 


### Reviews and Book Notices.

---

ARTICLE XX.—TRANSACTIONS OF MEDICAL SOCIETIES.

1. *Transactions of the American Ophthalmological Society.* Fourteenth annual meeting, held at Newport, R. I., 1878. Vol. II., Part 4.

2. *Transactions of the American Otological Society. Eleventh Annual Meeting.* Newport, 1878. Vol. II., Part 2.

1. The transactions of this society are presented in a modest pamphlet of 150 pages. As usual, there is but little chaff in the published proceedings of this society. Almost every page teems with new and interesting matter. This example of thorough winnowing is worthy of imitation by other societies.

First following the minutes of the meeting is a portrait or brief sketch of the life of Dr. H. Althof, one of the founders of the Society, who died Jan. 15, 1877.

Dr. A. Mathewson contributes "Notes on the Use of Nitrous Oxide as an Anæsthetic in Strabismus Operations." He thinks this agent offers special advantages as an anæsthetic in these operations over chloroform or ether, in that its effect so speedily passes away. In five cases he reports in which nitrous oxide was used, anæsthesia was produced in from one to two minutes and consciousness returned almost immediately. From seven to twenty gallons of liquid gas were used at a maximum cost of one dollar.

The relations of blepharitis ciliaris to ametropia receives further attention from Dr. Roosa. Since reading his first paper on this subject, in 1876, 201 cases of blepharitis have been observed at the Manhattan Eye and Ear Hospital, but in only 48 was the state of the refraction noted. In these, hypermetropia was found in 34, myopia 1, astigmatism 7, emmetropia 6. He has seen 46 cases of blepharitis ciliaris in private practice, in which there was found hypermetropia 25, astigmatism 17, myopia 2, emmetropia none. The refraction was not determined in two cases.

These statistics are remarkable, and we agree with the doctor that they "are certainly very different from those given by Dr. Alt," and we might add from the experience of others. However, we do not seriously object to the conlusions of Dr. Roosa, and do not see that they are severely antagonized by Dr. Hotz's paper,* which Dr. R. refers to as an answer to his (Roosa's) first paper.

Dr. J. S. Prout has a good article on "Lachrymal Conjunctivitis and some of the other Injurious Effects of Retention of the Tears." We are convinced that obstruction to the tears is a factor too often overlooked or disregarded in the treatment of conjunctivitis, and heartily endorse this article.

Dr. C. R. Agnew contributes an interesting paper on "Cases of Ophthalmic Disease in which Enforced Exposure to Light and Air was Salutary."

A practical and rapid method, with an instrument, for the diagnosis of refraction is presented by Dr. Wm. Thomson.

We are unable to pass upon the merits of the method or instrument from simply reading the description of them.

Dr. John Green also offers some improvements in instruments and appliances for diagnosis: 1. "Test Diagrams for the Detection and Measurement of Astigmatism." 2. "Stereoscopic Diagrams for Testing Binocular Vision." 3. "A New Modification of Loring's Ophthalmoscope." 4. "Improved Series of Arrangements of the Glasses of the Trial Case for Measuring Refraction."

Dr. E. G. Loring presents still another modification of the ophthalmoscope.

Dr. David Webster has an article on the "Ætiology of Retinitis Pigmentosa, with Cases."

Of the 22 cases presented, consanguinity of parentage was traceable in only 3, while heredity was present in 7 cases.

---

* Published in the JOURNAL AND EXAMINER, April, 1878.

Interesting cases are presented by Drs. Strawbridge, Bull, Vennyne, Derby, Dixon and others.

Numerous wood cuts, illustrating the text, add to the value of of these transactions.

2. "The Transactions of the Otological Society for 1878," is a neat pamphlet of 280 pages. More than 200 of these pages are filled with the interesting and exhaustive report on the progress of otology by Drs. Vennyne, of Massachusetts, and Sexton, of New York. This report is unusually long from the fact that there was no meeting in 1877, and it covers a period of 2 years.

First following this is a short article by Dr. C. J. Blake on "Acupuncture and Drainage in the Treatment of Serous Effusion into the Tympanic Cavity." The object in view is to avoid the irritation of the discharge upon the inner extremity of the auditory canal in those cases requiring paracentisis of the membrana tympani.

Drainage is effected by means of the absorbent cotton.

There may be exceptional cases in which this treatment would be worth the trouble, but we do not think the irritation from a serous discharge would be sufficient to demand it often.

Interesting cases are reported by Drs. J. Orne Green, A. H. Buck, C. R. Agnew, Geo. Strawbridge, A Mathewson and others.

Dr. Sexton has also an interesting article on "The Relations of the Conducting Mechanism of the Ear to Abnormal Hearing." He concludes that "Two distinct conditions appear to contribute to the abnormities of hearing: disarticulation of the malle-incudal joint and alterations in the tension of the membrana tympani." We agree with the doctor when, in speaking of the inner ear, he says, "Too great importance has perhaps been given the affections of this portion of the ear, especially as regards the acoustical anomalies." But it is certainly taking extreme ground in the other direction to relegate all abnormities of hearing to the conducting mechanism.

Dr. J. Orne Green has an article on "Objective and Subjective Systolic Murmurs in the Ears." His conclusions are that these murmurs may be divided into three classes:

Physiological murmurs in the internal carotid, dependent on partial stenosis of the carotid canal.

Pathological murmurs in the internal carotid, probably caused by a reduced vascular tension dependent upon disturbances in the vaso-motor system.

Pathological murmurs from aneurisms of the vessels of the head. W. T. M.

ARTICLE XXI.—THE CELL DOCTRINE; ITS HISTORY AND PRESENT STATE. By James Tyson, M.D., Professor in General Pathology and Morbid Anatomy in the University of Pennsylvania. Second edition, illustrated. Philadelphia: Lindsay & Blakiston.

The second edition of this well-known monograph issues from the press of L. & B. in elegant form. Readers of the first edition will find in this a large addition to the previously copious bibliography, and the incorporation and adaption of Flemming's observations (with Klein's indorsement) on intra-cellular structure. The tracing of the history and development of the cell doctrine is also somewhat more elaborate—we had almost said tedious—for we think that the value of the book would be enhanced in the eyes of nine-tenths of its readers if less space were given to the resurrection of dead and buried theories—some of them scarcely relevant, and more to the application of the prevalent theory to the facts of physiology and pathology.

The author credits Schleiden (1838) with the conception of the cell as the unit of structure, and Schwann with the successful application and demonstration of this conception in animal tissues; Martin Barry (1840) with a demonstration of cell-genesis by division of the nucleus; Max Schultze (1861) with having overthrown the vesicular idea of cells previously popular; and with identifying cell-contents with "sarcode." He credits Flemming with observations on intercellular structure, but neglects to mention Hensen's previous work in the same line.

Prof. Tyson thinks, however, that the present cell-doctrine was born of Beale, or rather, he adopts that born of Beale as the present cell-doctrine, and elaborates at length Dr. Beale's views on germinal matter and formed material. We notice with pleasure that the author deprecates the use of the term protoplasm, because of its ambiguity—being applied by some to the germinal or living matter, by others to the non-germinal or formed material. He proposes the substitution for protoplasm of Beale's name "bioplasm," which is not now, and from its derivation can never be, similarly misapplied.

He would further substitute "non-germinal" for Beale's "formed" material—an objectionable change, which should and will fail of general adoption. He indorses Auerbach's views that nucleoli are proven, by their amœboid movements and by their reactions toward staining agents, to be daughter cells; he thinks that a nucleus—i. e. a *differentiation* of germinal matter—is not essential to a cell; accepts Beale's interpretation of intercellular substance—that it, like the formed material proper, is transformed germinal or nuclear matter; and frowns mildly upon spontaneous generation and Charlton Bastian.

While, therefore, the book has a certain interest as a historical memoir, it is chiefly valuable for its clear and concise statement of Beale's views, and for its copious references to the literature of this and kindred subjects. W. J. B.

ARTICLE XXII.—HUMAN OSTEOLOGY, Comprising a Description of the Bones, with Delineations of the Attachments of the Muscles, the General and Microscopic Structures of Bone, and its Development. By Luther Holden, F. R. C. S., etc. Fifth Edition, Revised by the Author, with the Assistance of Alban Doran, F. R. C. S., etc. Philadelphia: Lindsay & Blakiston, 1878; Chicago: Jansen, McClurg & Co.; 8vo pp. 280. Price $5.50.

Many of our readers probably are acquainted with this excellent book, and all undoubtedly know the name of Luther Holden as an anatomist and author of valuable works, of which his Human Osteology is neither the last in rank nor the least in merits.

The present edition is equally as complete as the former, and, therefore, it is to be regretted that in one instance its perfectness should be marred by the oversight of the proof-reader. It has escaped his attention that all the numbers in the text referring to the figures on Plate II. are wrong; for instance, the text refers to fig. 4 as a transverse section of bone, while fig. 4 represents a longitudinal section; the text points to fig. 3 as illustrating the longitudinal section of bone, while fig. 3 represents cellular cartilage. This confusion of numbers in text and plate cannot fail to perplex the fresh students of anatomy, for whom the book has been written. Fortunately, however, under each figure on the plate is printed what it intends to represent, so that the errors in the text become apparent at once also to the uninitiated student of anatomy.

In the make-up of this book we notice the publishers have imitated the English style of binding the books with the leaves untrimmed. We never admired this, what seems to us, half-done style of book-binding; we do not like to read with a paper-cutter in the hand and we do not like to see a book in our library which looks as if every second leaf were coming out. We would suggest that the publishers adhere to their old fashion of sending out their books in that well-trimmed, elegantly-finished form for which American publications are admired all the world over. F. C. H.

ARTICLE XXIII.—THE ANTAGONISM OF THERAPEUTICS AND WHAT IT TEACHES. By J. Milner Fothergill, M.D., Edinburg.

In this little book, which is the essay to which was afforded the Fothergillian Gold Medal of the Medical Society of London, for 1878, Dr. Fothergill has given us in a concise form the most important results of the experiments which have been made to ascertain the physiological antagonism of an important list of medicines. This he does by detailing the experiments; by a practical inquiry relative to the experiments performed; by a discussion of the rhythmically discharging centers; by a discussion of the action of drugs on the circulation and respiration; the use of antagonisms in actual poisoning and in ordinary practice. We have no hesitation in saying that this little book will contribute largely, based as it is on such well-authenticated experiments, towards removing the stigma of empiricism which is so often but justly applied to a great deal of general practice. To show the practical bearing of the work we quote from the chapter on antagonism in actual poisoning. After detailing a case of poisoning by about seventeen grains of opium, Dr. F. says:

"If a case of opium poisoning were to present itself to me again, especially at an early stage, where I could carry out my ideal plan of treatment, it would go as follows: 'First, empty the stomach thoroughly, then inject a fourth or a third of a grain of atropine before the respiration is gravely affected; then put the patient to bed and carefully note the respiration, the pulse and the temperature. If the respiration were still failing after the administration of the atropine, to inject another third of a grain and still take careful notes, and give a third third of a grain, or even more, if the respiration still fell.'"

The chapter which treats of the action of chloral cannot but enlarge the views of all who read it. R. T.

ARTICLE XXIV.—ESSENTIALS OF CHEMISTRY, INORGANIC AND ORGANIC, FOR THE USE OF STUDENTS IN MEDICINE. By R. A. Whitehouse, A.M., M.D. New York: Wm. Wood & Co.

This little book, which can readily be carried in the pocket, consists of a series of questions and answers, and will form an excellent supplement to the general text book on chemistry. Some people pretend to disdain these small books, which serve in great part the purpose of a more expensive "coach." So far, however, as our experience has gone, those who have used them have always shown best in a case of examination, and their knowledge has not been the more shallow for the assistance thus afforded them. The series of questions on the compounds of carbon constitute an important feature of the book. For those fond of chemistry, who are so situated that they cannot obtain a teacher, this book will be a boon companion. For those who have a teacher it will be a valuable aid. R. T.

ARTICLE XXV.—ELEMENTARY QUANTITATIVE ANALYSIS. By Alexander Clanen, Professor in the Royal Polytechnic School, Aix-la-Chappelle. Translated with additions by Edgar F. Smith, A.M., PH.D.. Cloth, pp. 328. Phila.: Henry C. Lea. Chicago: Jansen, McClurg & Co.

The translation of this brief treatise on quantitative analysis will be found of benefit alike to professors and students who are not familiar with the original language in which it is written

The most approved methods of determining the different elemental and primary compound substances are clearly and concisely displayed, and the suggestions which the experience of the author has found desirable, constitute an important feature of the work.

R. T.

ARTICLE XXVI. ST. BARTHOLOMEW'S HOSPITAL REPORTS. Vol. XIV., 1878. Edited by W. S. Church, M.D., and Alfred Willet, F.R.C.S.

It is but a short time since we had the pleasure of noticing the "Reports" for 1877. The volume under present consideration is in no way inferior to it. Exception can hardly be taken to a single paper of the thirty-three contributed. Without attempting to notice each paper, we must make speical mention of the following: "Observations on Acute Rheumatism," by Dr. Southey; "On the Varieties of Icterus Gravis," by Dr. Legg. Sir James Paget has an admirable article on "Indurations of the Breast becoming Cancerous." Mr. Hussey reports a number of cases of Hydrocele, his favorite treatment being the seton, which in his hands has seemed very useful. Dr. Matthews Duncan reports a case of "Uterine Fibroid Complicated with Inversion;" and Mr. Lyons has an illustrated paper on the Treatment of Compound Fractures of the Lower Jaw, in which he favors the "wire interdental splint." Mr. Doran continues a former discussion on "Intra-peritoneal Ligature of the Pedicle in Ovariotomy," for which he puts in a well-considered plea.

Dr. Moore and Mr. Callender favor us with their experiences respectively, in the casualty and surgical departments. One of the most interesting papers in the volume, in a certain sense, is the "Account of the Casualty Department," given by Mr. Bridges. The casualty department is simply the vast hospital dispensary where some 200,000 patients are annually treated. He gives an admirable idea of the amount of work done by the staff, of the expense, and the amount of benefit actually rendered; and, on the whole, the paper will amply repay for reading by those connected with our smaller institutions of a similar character.

In a paper on "Manipulation," Mr. Marsh has a word to say about the bone-setters and some of their results, giving cases. Mr. West follows with an "Analysis of Forty Cases of Rheumatic Fever," and Dr. Church with a very carefully reported "Case of Intra-thoracic Tumor." Dr. Harris has an interesting paper on the "Diagnosis and Treatment of Apparent Drunkenness," in which he shows how easy it may be to mistake the alcoholic for the apoplectic condition, and vice versa. He thinks it better to treat all such cases very carefully in order to be on the safe side.

Mr. Ormerod contributes some "Cases of Diseases of the Spinal Cord," and Mr. Steavenson some "Observations on the action of Pilocarpine" in three cases of Bright's disease, in each of which the drug had to be discontinued. Mr. Cripps adds some statistics with regard to the heredity of cancer; and Mr. Keetley a paper on "Compression versus Inflammation," in which he argues that the great advantages of properly applied compression are overlooked and under-estimated.

In the way of literature of special departments we are favored with papers by Mr. Clarke on "Wounds of the Ciliary Region," and by Mr. Vernon on "Commencing Cataract." Both of these are admirable. The series of papers closes with two papers by Mr. Willett on "Spinal Disease."

The "Proceedings of the Abernethian Society," and the usual statistical tables complete this fourteenth volume, which is replete with interesting matter. R. P.

ARTICLE XXVII. — CYCLOPÆDIA OF PRACTICE OF MEDICINE. Edited by Dr. Von Ziemssen. Vol. VIII. Diseases of the Chylopoëtic System, with Chapters relating to Diseases of the Bladder and Urethra and Male Genital Organs. Wm. Wood & Co., New York, 1878.

This volume includes articles by Ziemssen and Zenker on Diseases of the Œsophagus, including morbid growths, parasites, etc., covering 214 pages.

Bauer devotes 130 pages to Diseases of the Peritoneum, including typhlitis and perityphlitis. Mosler's contribution on Diseases of the Spleen covers 200 pages, and includes leucæmia and melanæmia.

Concerning Diseases of the Pancreas, of which little or nothing can be diagnosed during life, Friedreich gives us 80 pages; while Merkel adds nearly 40 pages on Diseases of the Supra-Renal Capsules, an equally obscure subject. With reference to the important topic of Bladder and Urethral Affections, Lebert furnishes an article of 150 pages, and Curschmann supplements it with nearly two-thirds as many pages on Diseases of the Male Genitals. We have been particularly pleased with the stand taken by the last two writers concerning the delicate but important topics of masturbation, seminal losses or spermatorrhœa, false and true, and the pages on chronic pyorrhœa in females, and pyorrhœa, both inflammatory and chronic, in males.

The volume is a very important one, and contains a vast store of information gathered from various sources in a very interesting and condensed shape. P.

Article XXVIII.—Cyclopædia of Practice of Medicine. Edited by Dr. Von Ziemssen. Vol. XIII. Diseases of the Medulla Oblongata and Spinal Cord. By Prof. Erb, of Heidelberg. New York, Wm. Wood & Co., 1878.

The volume opens with an introduction, anatomical and physiological, followed by general symptomatology and ætiology, and general therapeutics. This portion alone, from such a master-hand, is of great value. We are pleased with his views, coinciding, as they do, with those of the best specialists, that masturbation is not, *per se*, more pernicious than natural coitus.

Diseases of the Membranes of the Cord require 70 pages for their elucidation, while 540 pages are devoted to diseases of the cord itself. Under this latter head are included wounds, spinal concussion, spinal irritation, which the author recognizes as a distinct condition, and spinal nervous weakness.

Myelitis, Acute and Chronic, the Various Forms of Sclerosis and Paralysis, and "Rare and Doubtful Diseases" receive very extended consideration.

The balance of the volume is devoted to Diseases of the Medulla; this part opening with another introduction to this particular subject, equally elaborate with the rest of the author's work. Hyperæmia, anæmia, injuries, acute bulbar myelitis, bulbar paralysis, and various other lesions are handled in a most scholarly way. We are inclined to think the author lays too much stress on the diagnostic value of the tendon-reflex, but this is a comparatively trifling matter. The book is of immense value, and should and does stand a monument to the indefatigable industry of its author. P.

